# Gender, homelessness, hospitalization and methamphetamine use fuel depression among people who inject drugs: implications for innovative prevention and care strategies

**DOI:** 10.3389/fpsyt.2023.1233844

**Published:** 2023-11-01

**Authors:** Lionel Moulis, Sao Mai Le, Vinh Vu Hai, Duong Thi Huong, Khuê Pham Minh, Khuat Thi Hai Oanh, Delphine Rapoud, Catherine Quillet, Tuyết Thanh Nham Thi, Roselyne Vallo, Giang Thi Hoang, Jean-Pierre Moles, Didier Laureillard, Jonathan Feelemyer, Don C. Des Jarlais, Laurent Michel, Nicolas Nagot, the DRIVE Study Team

**Affiliations:** ^1^PCCEI, University of Montpellier, INSERM, EFS, University of Antilles, Montpellier, France; ^2^Haiphong University of Medicine and Pharmacy, Haiphong, Vietnam; ^3^Department of Infectious and Tropical Diseases, Viet Tiep Hospital, Haiphong, Vietnam; ^4^Supporting Community Development Initiatives, Hanoi, Vietnam; ^5^Infectious Diseases Department, Caremeau University Hospital, Nîmes, France; ^6^School of Global Public Health, New York, NY, United States; ^7^CESP Inserm UMRS, Pierre Nicole Center, Paris Saclay University, Fench Red Cross, Paris, France

**Keywords:** mental health, mood disorder, substance abuse, Vietnam, PWID, depression, prevention, dual diagnosis

## Abstract

**Background:**

The co-occurrence of substance use disorder and mental disorder, known as dual diagnosis, has a distressingly high prevalence among individuals grappling with either of these conditions. Mood disorders, especially depression, constitute a substantial burden for People Who Inject Drugs (PWID) and a significant public health concern in Vietnam. Identifying risk factors for depression in PWID is imperative for the development of targeted interventions.

**Methods:**

We enrolled PWID into a cohort using the respondent-driven sampling method. Over a 36-month period, we systematically tracked the emergence of depression and employed multiple imputation in conjunction with a mixed nonlinear model to pinpoint risk factors for depression in this demographic. At inclusion, depression was screened using the PHQ-2 questionnaire, and subsequent episodes of depression were assessed semi-annually using the CES-D8.

**Results:**

Three hundred and ninety-one PWID (26.6%) were depressed. Major risk factors for depression included being female, not having a permanent residency, having been hospitalized and using methamphetamine more than weekly. Other risk factors included age, being single, not having a health insurance card and not being on methadone.

**Limitations:**

The exclusion of missing visits and social desirability could have led to selection and information biases. In this observational study, confusion biases are possible despite our best efforts.

**Conclusion:**

Depression is alarmingly frequent in PWID. In this study taking in account the chronological relationship between sociodemographic and clinical factors and depression, risk factors were identified in this specific setting of low-to-middle income country. The findings highlight the need to develop innovative targeted psychiatric interventions with the help of supporting peers.

## 1. Introduction

Dual diagnosis, or the co-occurrence of substance use disorders (SUD) with psychiatric comorbidities (such as depression, anxiety disorders, suicidal tendencies, etc.), is relatively uncommon in the general population (approximately 2 to 3%) (1). However, it is found in an alarming prevalence ranging from 40 to 50% in patients presenting with either condition (2–5). While further elucidation is required to fully comprehend the reasons behind the frequent association of these two conditions (6, 7), it is observed that they tend to progress concurrently within the same individual, exerting mutual influence on each other’s prognosis (8). Consequently, they lead to severe consequences in socio-professional aspects (9), clinical domains (physical comorbidities, risk behaviors) (10–13), delayed detection of viral illnesses (14), and hindered access to healthcare in general (15).

The most common psychiatric condition in patients with SUD are mood disorders, with prevalence rates varying from 17% in Canada to a broader range of 27 to 85% in Australia, as reported in a meta-analysis (1, 4). More specifically, the prevalence of major depressive disorders in community and clinical settings among individuals with SUD was found to be 25% in a comprehensive meta-analysis comprising 48 studies from around the world (16). However, these rates varied depending on the studied population, type of substance and country considered.

In Vietnam, as in numerous low- to middle-income countries (LMICs), psychiatric resources are sparse, resulting in a saturated mental health system (17) managing mainly the most severely ill patients because of a lack of human and material resources.

In this context, targeting specifically people who inject drugs (PWID) who are in need of mental health support is therefore crucial for they present, among other mental health issues, significantly higher rates of depression (18). Previous studies analyzing risk factors for depression were sparse among PWID living in LMIC (19), where the expression of depression is not as prevalent as in high income countries and where associated factors might differ. Moreover, these studies with cross-sectional designs (20) identified only factors associated with depression and not risk factors for depression whose estimating requires following up the population (21, 22). Indeed, the chronological knowledge of the situation must be taken in account in order to better understanding the mechanisms and offer tailored prevention strategies.

The aim of our project is to develop a community-based innovative intervention plan to prevent and limit the burden of depression in this specific population. In order to do so, we aimed in this study to explore longitudinal associations between depressive symptoms and patient characteristics in people who inject drugs in Hai Phong, Vietnam.

## 2. Materials and methods

### 2.1. DRIVE study population and recruitment

The Drug use and Infections in Vietnam (DRIVE) program is an international project launched in 2016 whose goal was to end the HIV epidemic among PWID in Hai Phong, Vietnam (23). The DRIVE study relied on extensive HIV screening *via* community-based respondent-driven sampling (RDS) surveys combined with linkage to HIV care. The RDS method allows access to hard-to-reach populations, hidden from care and inaccessible through classic recruitment methods (24).

Three rounds of annual RDS surveys from November 2016 to November 2018 targeted 3,150 PWID. The inclusion criteria were being at least 18 years old, having a positive urine test for heroin or methamphetamine and having skin marks from a recent injection (23). Those unable to understand the study information or unwilling to consent were not enrolled.

Starting from the first RDS survey, two open cohorts were created: one with HIV-negative participants to estimate HIV incidence and another with HIV-positive participants to estimate the cascade of HIV care dynamics. All RDS HIV-positive participants were invited to be included in the cohort and were then provided counseling and peer support to help them connect with care and initiate or remain on antiretroviral treatment (ART) as well as encourage them to pursue methadone maintenance therapies (MMTs). HIV-negative participants were recruited until the target cohort size was reached, which was set to 400 for RDS1 and 400 for the next two RDS rounds, excluding those who reported already being treated with methadone (25). Participants were enrolled in the cohort for 12 to 36 months depending on their date of recruitment, with follow-up visits every 6 months.

### 2.2. Selection

In this study, sociodemographic, drug habit, psychological, behavioral and health event-related data from all PWID recruited in the previously described HIV+ and HIV- cohorts and who participated in at least one follow up visit were included.

### 2.3. Measures

#### 2.3.1. Primary outcome

At baseline (RDS survey), depression was screened with a short form of the Patient Health Questionnaire (PHQ-2) (26), in face-to-face interview with trained community-based organization (CBO) member. Participants with a score strictly superior to 2 were considered depressive (27, 28).

During semiannual follow-up visits, depression was measured by trained doctors from the Hai Phong University of Medicine and Pharmacy using a short form of the Center for Epidemiologic Studies- Depression (CES-D8) questionnaire (29), shown to facilitate the efficiency of assessment during epidemiological studies (30). Because the original cutoff of ≥7 was determined in women, who represent 5% of PWID in Hai Phong, we used a lower cutoff of ≥6 (31).

#### 2.3.2. Other psychiatric disorders

Participants were screened for anxiety at the time of the RDS survey with the short Generalized Anxiety Disorder questionnaire (GAD-2) developed to screen for anxiety in primary care patients and the general population, with a published cutoff of ≥3 (32).

Participants who screened positive for any mental health issue during the RDS survey or during follow-up were invited to meet with a psychiatrist for a full clinical assessment followed by other consultations when necessary at the study site.

#### 2.3.3. Substance use

Data were collected by trained interviewers (CBO members) using a face-to-face questionnaire on drug and alcohol use. The questionnaire created for the study included questions on duration, age of initiation, frequency and substances injected, as well as on the use of other non-injected substances including alcohol. Its content has been described in greater detail elsewhere (25, 33). PWID were also screened for opiates, methadone, methamphetamine and cannabis by urine tests.

A *high heroin injection frequency* referred to PWID reporting more than 30 injections in the past month. *Methamphetamine use* had two categories, “no use or less than once per week” and “more than once per week” (34). We combined urine tests and questions about drug use in the past 6 months to define *polydrug use* as using any drug other than heroin or methamphetamine (ketamine, ecstasy, cocaine or cannabis) in addition to heroin, which was used by all participants.

*Alcohol misuse* was assessed through Audit-C, a 3-item questionnaire including the first questions of the AUDIT questionnaire, with a cutoff of ≥3 for women and ≥ 4 for men (35).

#### 2.3.4. Other medical data

At the RDS visit, CBO members collected individual sociodemographic measures: *gender, age* and *education level*. During the cohort follow-up, they collected data on major health and life events (*overdoses* and *arrests*), as well as social and structural sociodemographic measures (*relationship status, stable housing* and *health insurance card*). PWID were also asked if they had *been hospitalized* during the past 6 months, whatever the reason, and were tested for HCV and HIV at every visit.

### 2.4. Statistical analysis

We analyzed risk factors for depressive syndrome among PWID over up to 3 years. These potential risk factors were sociodemographic (*gender, age, relationship status, stable housing* and *health insurance card*), drug habits (*methamphetamine use, high heroin injection frequency, polydrug use, alcohol misuse, being on MMT* and *more than 10 years since the first heroin injection*), and health events (*having been hospitalized in the past 6 months, HCV status* and *HIV status*). Anxiety was not entered in the model as cofounder because of anxiety-related questions within the CES-D scale (36). People with depressive syndrome at baseline according to the PHQ-2 were not included in our model. We censored participants from the time they began antidepressant treatment.

A mixed nonlinear model was used for the main analyzes in order to explore longitudinal associations between depression and patient characteristics. We developed multiple models that differed in the number of variables and the inclusion of random intercepts and then compared their quality to select the final model.

To deal with missing completely at random (MCAR) data for participants who had completed their follow-up visits, we selected complete outcome cases and used a multiple imputation technique: the model was apply to 20 datasets of possible values created *via* linear regression or discriminant function method depending on the variable and parameter estimates were combined to obtain final adjusted odds ratio and their 95% confidence intervals (CIs) (36–38).

The data analysis for this paper was performed using SAS software version 9.4. Copyright © 2013 SAS Institute Inc.

### 2.5. Ethics consideration

The DRIVE study was approved by the Institutional Review Boards of the Hai Phong University of Medicine and Pharmacy, the Icahn School of Medicine at Mount Sinai, and the New York University School of Medicine. All participants provided signed informed consent.

## 3. Results

### 3.1. Population selection

**Figure 1:**
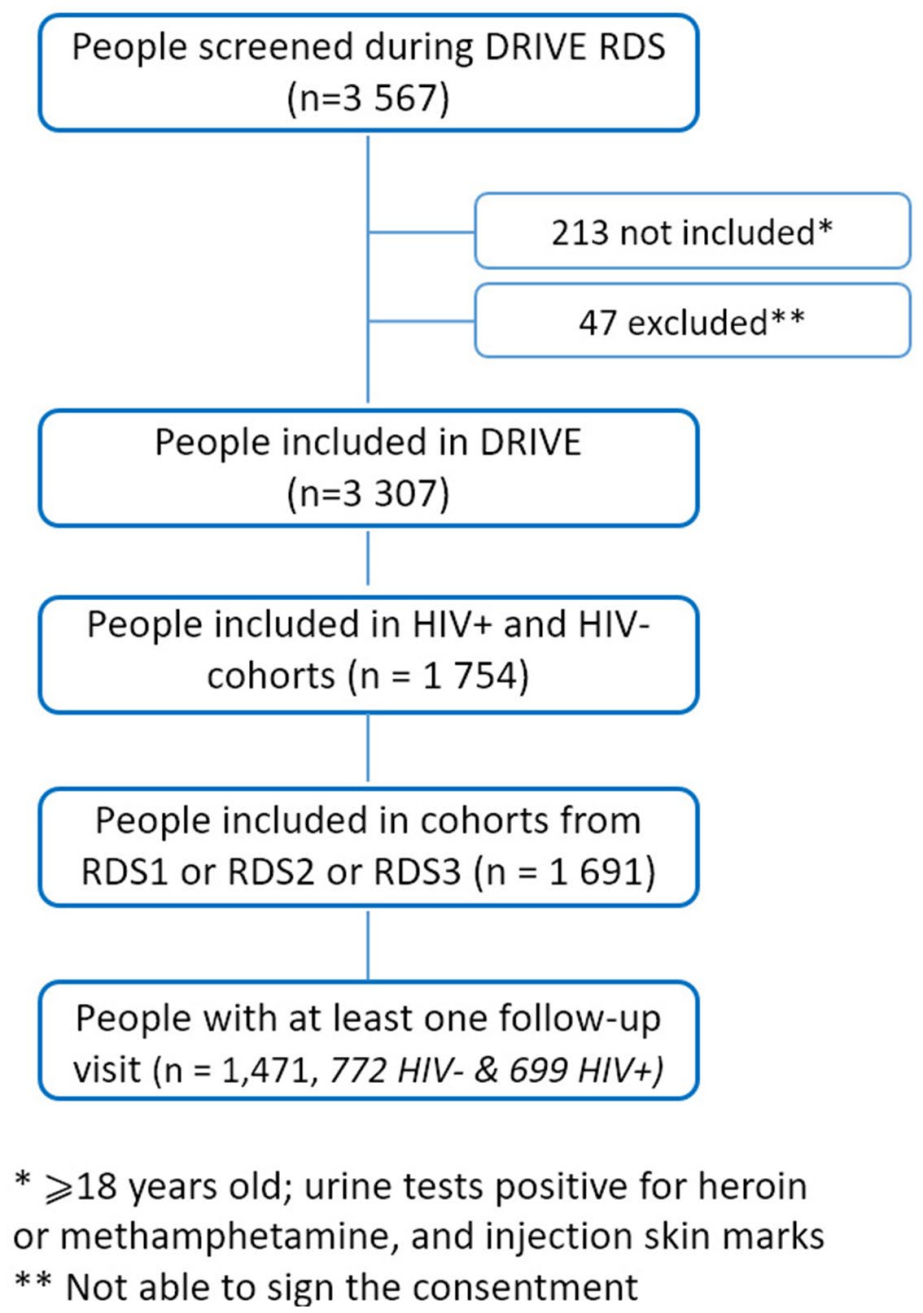
Flow chart (simplified).

Of the 1,691 people included in the DRIVE cohort, 215 never returned for any follow-up visits, 2 died. and 3 began antidepressant treatment before the first follow-up. In total, 1,471 people were included in our study, 772 from the HIV-negative cohort and 699 from the HIV-positive cohort (Figure 1). They were followed up from 2 to 35 months (16 months on average) with a mean number of 3.3 visits. 771 PWID followed up during 6 visits, 371 during 4 visits and 329 during 2 visits were included at the first, second and last RDS survey, respectively (Supplementary Figure S1).

### 3.2. Population characteristics

All participants were heroin users, 93% were male, and their mean age was 41 years (Table 1). Overall, HIV-infected participants were less educated (53% vs. 70%), injected heroin for longer (63% used it for more than 10 years vs. 37%), and were more often on MMT (35% vs. 12%).

**Table 1:** Characteristics of the population at inclusion.

Characteristics	HIV– (*n* = 772)	HIV+ (*n* = 699)	Total (*n* = 1,471)
Sociodemographic			
Male	710 (92.0)	659 (94.3)	1,369 (93.1)
Female	61 (7.9)	39 (5.6)	100 (6.8)
Transgender	1 (0.1)	1 (0.1)	2 (0.1)
Age	40.9 (± 9.4)	40.4 (± 6.5)	40.6 (± 8.1)
At least high school level	541 (70.1)	371 (53.1)	912 (62.0)
In a relationship	325 (42.1)	250 (35.8)	575 (39.1)
Income ⩾ 6 VND^1^	353 (45.7)	258 (36.9)	611 (41.5)
Stable housing	744 (96.4)	680 (97.3)	1,425 (96.8)
Health insurance card	285 (36.9)	463 (66.2)	748 (50.9)
Sex work	23 (3.0)	9 (1.3)	32 (2.2)
Psychiatric data			
Anxiety	72 (9.3)	50 (7.1)	122 (8.3)
Depression (PHQ-2)	74 (9.6)	58 (8.3)	132 (9.0)
Suicidal ideation (past 2 weeks)	102 (13.2)	76 (10.8)	178 (12.1)
Drug habits			
Alcohol misuse (AUDIT-C)	315 (40.8)	260 (37.2)	576 (39.1)
Methamphetamine > once a week	166 (21.5)	101 (14.4)	267 (18.1)
Recent methamphetamine use^2^	528 (68.4)	439 (62.8)	967 (65.7)
Polydrug use^3^	121 (15.7)	97 (13.9)	218 (14.8)
Years of heroin injection			
< 5 years	212 (27.4)	43 (6.1)	255 (17.3)
5–10 years	210 (27.2)	158 (22.5)	368 (24.9)
10–15 years	174 (22.5)	180 (25.6)	354 (24.0)
> 15 years	177 (22.9)	321 (45.7)	498 (33.7)
Age at first heroin injection	31.4 (± 8.9)	27.3 (± 7.3)	29.4 (± 8.4)
High-frequency heroin use	603 (78.1)	501 (71.7)	1,109 (75.1)
Methadone Maintenance therapy	93 (12.0)	242 (34.6)	335 (22.8)
Cannabis (past 6 months)	103 (13.3)	123 (17.6)	226 (15.4)
Cocaine(past 6 months)	5 (0.6)	9 (1.3)	14 (0.9)
Ketamine (past 6 months)	43 (5.6)	28 (4.0)	71 (4.8)
Ecstasy (past 6 months)	40 (5.2)	30 (4.3)	70 (4.8)
Amphetamine (past 6 months)	124 (16.1)	95 (13.6)	219 (14.9)
Urine tests			
Heroin	768 (99.5)	697 (99.7)	1,465 (99.6)
Methamphetamine	271 (35.1)	213 (30.5)	484 (32.9)
Cannabis	47 (6.1)	205 (29.3)	252 (17.1)
Cocaine	0 (0)	0 (0)	0 (0)
Ketamine	0 (0)	0 (0)	0 (0)
Methadone	207 (26.8)	448 (64.1)	655 (44.5)
Infection status			
Undetectable HIV RNA^4^	NA	490 (70.1)	NA
HCV-positive serology	445 (57.6)	611 (87.0)	1,053 (71.6)

^1^Equivalent to ~ 260 USD per month; ^2^In the month; ^3^Cannabis, cocaine, ketamine or ecstasy; ^4^Undetectable or viral load lower than 20 copies/mL. The results are given as number (percentage) or mean (± standard deviation).

Of our 6,768 planned follow-up visits, 5,201 (76.8%) were completed. During follow-up, 56 people had died, including 1 from suicide, 2 from overdoses and 2 for unknown reasons. Other causes of death had no direct link with depression. 394 (5.8%) visits were attended but had incomplete data on the CES-D8 questionnaire.

The 329 participants who dropped out (22%) were younger, were less likely to be in a relationship or in a stable housing, were more depressed at inclusion, and were more likely to use methamphetamine more than once per week. They were less likely to be enrolled in methadone maintenance therapy and less likely to be infected by HIV (Supplementary Table S1).

### 3.3. Prevalence of depression

The proportion of depressed participants was 11.9% at baseline. The number of PWID depressed at one or more visits was 391 (26.6%). Among people free from depression in the 2 weeks prior to inclusion based on the PHQ-2, 320 (23.8%) developed depression over the mean 16 months of follow-up.

### 3.4. Risk factors for depression

**Figure 2:**
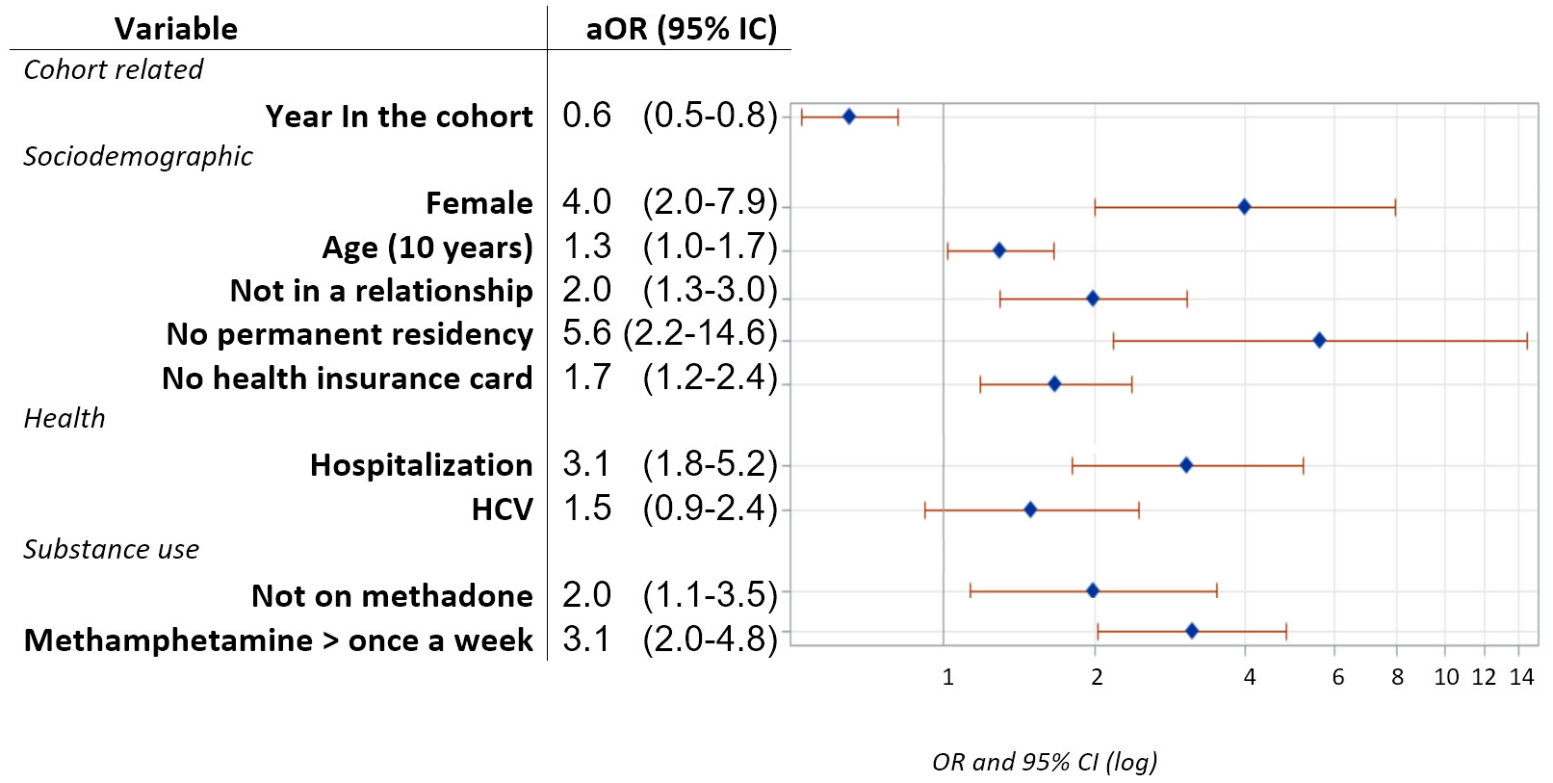
Adjusted odds ratios and their 95% confidence intervals for factors associated with depression obtained using a nonlinear mixed model with a cutoff CES-D8 score ⩾ 6 for people free from depression according to the PHQ-2 data at inclusion.

No permanent residency [OR = 5.6, 95% CI (2.2–14.6)], gender [female: OR = 4.0, 95% CI (2.0–7.9)], having been hospitalized and using methamphetamine at least once a week [OR = 3.1, 95% CI (1.8–5.2)], not being in relationship nor on methadone [OR = 2.0, 95% CI (1.1–3.5)], not having an insurance card [OR = 1.7, 95% CI (1.2–2.4)], age [OR = 1.3, 95% CI (1.0–1.7) for every 10 years] were all significant risk factors for depression. Time since inclusion in the cohort was a protective factor against depression [OR = 0.6, 95% CI (0.5–0.8) for each year in the cohort]. Being infected with HCV tended to be associated with depression without statistically significant (Figure 2).

## 4. Discussion

### 4.1. Identifying PWID with a high depression risk

In this cohort of PWID recruited through RDS surveys, almost a quarter had depressive symptoms develop or reoccur during a follow-up of 16 months on average. This is more than previously found in a Canadian open cohort of PWID (39) but lower than in other PWID populations, whether in LMICs (40) or in high-income countries (41). This could be explained by the different methodologies (population selection, screening tools) used to measure the prevalence of depression. Furthermore, in Asian settings, the expressiveness of depressive symptoms has been shown to vary: depressive people from LMICs show fewer mood-related symptoms, which are what the CES-D8 mainly measures, and more somatic symptoms (42, 43).

The gender gap in depression has been extensively studied in the general population and could be attributed to various factors. Hormonal fluctuations (44, 45), differences in expressed genes and neural and cerebral circuitry architecture (44), personality traits (46, 47), societal factors such as the lower levels of education (48, 49), life stressors (50), and gender norms influencing answers of certain CES-D8 questions (51) could all be part of the explanation. Nevertheless, in this particular population of female PWID, the stigma associated with drug use could have a significant impact on depressive symptoms (52). This effect has been observed not only globally (53) but also in Vietnam, where female PWID experience higher levels of stigmatization, both from men and women (54), which may have concerning implications for their mental health (55).

Another potential explanation could be gender disparities in involvement in sex work. While we could not establish gender differences in sex work due to underreported or unanswered questions in this study, another article from the DRIVE project, focusing on a similar population, found that being a woman was linked to engagement in sex work (56), a known risk factor for depression (57). Although reaching sex workers is difficult (fear of stigma, desirability biases, conditions of life, night work, pimp control, etc.), psychological interventions, screening for mental health in health care services targeting female sex workers, and training “gatekeepers” to support women at risk, should be implemented (58). More generally, targeted prevention efforts should consider that women may be at a higher risk of depression and offer them dedicated interventions.

Other factors reflecting social precariousness emerged. “Living on the edge” without stable housing or a health insurance card has already been identified as a risk factor for mental illness (59). The concerns and anxiety this causes could exacerbate or trigger the onset of mental illness, acting as additional stress on a potentially compromised state of mental resilience already affected by substance use. Similarly, social support, as defined in our study as being in a relationship, was a protective factor for depression, probably because of both its social and its emotional support impacts (60, 61).

The prevalence of depression increases with age in both the general population and PWID (62, 63). This may be explained by age-related biological changes and comorbidities or chronic conditions, all of which are strongly associated with depression (64). As we did not have data about comorbidities, it is possible that age acted as a proxy of chronic health problems (65). The association found between depression and hospitalization during follow-up, probably reflecting the decompensation of somatic comorbidities, is consistent with this hypothesis.

Indeed, having been hospitalized during the follow-up increased the risk of depression. Despite the fact that we had no information on the causes of these hospital admissions, they might be related to complications or treatments of infections, which are frequent in our cohort population, as well as a degraded state of health, commonly associated with depression (12).

We found that HCV had a tendency to increase the risk of depression, although not significantly. In literature, this observed association (66) has been described as linked to poor health-related quality of life and physical, mental and social illnesses due to the disease (67), the treatments (68), and even direct HCV neuro-invasion, and metabolic derangements (69). Nevertheless, as new cost-effective direct-acting antivirals for HCV have recently been developed with lower psychiatric side effects (70–72), we can see how they will become widespread, including among LMICs, thus reducing the mental health burden of HCV.

Generally, we observed that time since inclusion was a protective factor. Our patients participated in a research program offering them the opportunities to be tested, examined and treated. Furthermore, the positive social impact of being enrolled in a cohort and going to regular meetings with CBO-trained members for social support, harm reduction and social and administrative counseling might have helped reduce the risk of depression. Being part of a community also offers, in the context of layered drug- and HIV-related stigma, the possibility to disclose their situation to someone (73).

### 4.2. Taking care of PWID with a high depression risk

In many countries, where access to psychiatric care is notably limited, alternative and original strategies that rely on nonprofessional or peer workers could be crucial. Using simple screening tools in the community to introduce drug users to their mental health may be the first step.

A simplified psychiatric screening tool adapted to nonprofessional/CBO members was developed as part of the DRIVE project. This 9-item questionnaire validated among PWID with acceptable psychometric characteristics and good acceptability (74, 75) allows a discussion of mental health, depression, psychiatric consequences of regular drug use and their treatment with trained CBO members, particularly for PWID with multiple risk factors. Depending on the score, a consultation with a psychiatrist *in situ* is proposed. Screening followed by delocalized psychiatric consultation is one of the innovative approaches that allows the care and monitoring of hard-to-reach vulnerable populations.

Psychiatric complications, particularly depression but also psychotic manifestations, are very common among frequent methamphetamine users and need special attention and tailored harm reduction interventions (76). Adapted information on drug use and the benefits of reducing should be provided by peers or social workers. For users who want to adapt their use, they should be given the means to change his or her behavior, thus limiting the risk of depression (77, 78).

Enrollment in MMT should be encouraged, since having the easiest possible access to MMT was found to be a protective factor (41, 79–81). Methadone-induced biological stabilization protected the patients from craving phases and allowed them personal and social stabilization (82, 83). Regular opioid use could even have a protective effect against psychiatric comorbidities (84). Indeed, this enables a decline in risk practices and the patient to be in touch with care professionals (85). Of course, the decision to initiate a patient in an MMT program should be made by a physician, following a thorough assessment of contraindications and in agreement with the patient. This decision should be preceded by informed consent, which includes a discussion of the risks and benefits of such a program, as well as alternative treatments, to enable the patient to make an informed decision.

It has become necessary to promote the development of psychiatric interventions in environments dedicated to drug users (methadone or HIV clinics) (78). In this aim, basic training on the association between mental health and addiction is required for all stakeholders, which implies acceptance of delocalized psychiatric interventions in specialized settings or CBO offices (86). Reducing the stigma associated with drug use and mental health disorders is urgent, and recognizing mutual competencies and roles of peers and healthcare workers is crucial (87).

### 4.3. Study limitations

First, owing to limitations in resources, staffing, and organizational capacity, we were unable to conduct psychiatric evaluations based on international criteria. The PHQ-2 used at baseline has a sensitivity of 72% (28), meaning that we did not exclude some people with clinical depression. Moreover, the questionnaire relates only to symptoms in the past 2 weeks, which could have biased our selection of people without depression. Although the presence and severity of depressive symptoms are known to vary over time, we did not evaluate history of depression. Furthermore, the semiannual assessment of depression involved a different screening tool with different psychometric properties, the CES-D. This questionnaire focus on symptoms in the last week, and we use a short 8-item version. However, this version has a correlation of 0.93 with the complete scale (30). Additionally, in another part of the DRIVE project (not shown here), psychiatric evaluation and the CES-D8 were found to have good agreement, indicating that as imperfect as this outcome was, it still served as a suitable proxy for depression assessment. The limitations of using different questionnaires in the study are well acknowledged by the investigators but provide insight into the evolution of depressive symptoms in a context of participant attrition. If these limitations were to introduce biases, they would likely be of minor magnitude and should not undermine the findings.

Second, we had to restrict our data to people with at least one follow-up visit; some were lost to follow-up, and we considered only visits with completed outcomes. This could induce some selection biases because it is plausible that people with high depression risk are the least able to visit, meaning that the more severe cases of depression were not as represented as others. We observed this phenomenon when we compared people with missed or excluded visits with the rest; the former presented more risk factors for depression.

Third, sampling biases could occur because our recruitment method included a large majority of male PWID. Our findings are representative of Vietnam and South Asia (88) but might not be extrapolated to populations of PWID with a lower proportion of males (approximately 75% around the world) (89). Similarly, our cohort overrepresented HIV-positive participants (approximately half), whereas the estimated prevalence of HIV among PWID is 30% in Hai Phong (24). In our analyzes, HIV status had no association with depression, which may seem surprising, as the link between depression and HIV status has been shown (90). In our cohort, however, we speculate that HIV-positive participants are less depressed because of greater support and free access to care offered by the DRIVE project.

Finally, avoided sensitive questions and missing data could be explained by desirability biases. For instance, questions about heroin experience and sex work were often not answered. Recall bias could also have occurred since questions were related to past behaviors.

## 5. Conclusion

The co-occurrence of addiction and depression is a scourge with catastrophic consequences for individuals affected by it. In this large longitudinal study focusing on people who inject drugs in a low-to-middle income country setting, we found a concerning high prevalence of depression.

This work was able for the first time in this setting to consider the temporal relationships of the factors studied and their impact on depressive symptomatology. We found that being a woman was strongly associated with depression. Certain factors of social precariousness were also identified as risk factors, as well as the consumption of methamphetamine in addition to substance injection. The beneficial effect of being on a MMT program was also emphasized.

All these results drive, in the context of limited psychiatric resources, the development of innovative interventions focusing on these vulnerable sub-populations, such as simple screening tools, delocalized psychiatric consultations, peer support, free treatment, etc.… Other interventions remain to be designed and tested in these specific settings.

## Data availability statement

The original contributions presented in the study are included in the article/Supplementary material, further inquiries can be directed to the corresponding author.

## Ethics statement

The studies involving humans were approved by the Institutional Review Board of the Hai Phong University of Medicine and Pharmacy, the Institutional Review Board of the Icahn School of Medicine at Mount Sinai, and the Institutional Review Board of the New York University School of Medicine. The studies were conducted in accordance with the local legislation and institutional requirements. The participants provided their written informed consent to participate in this study.

## Author contributions

LiM: conceptualization, methodology, software, formal analysis, writing–original draft, and visualization. SL: investigation, validation, and writing–review and editing. VH: investigation and writing–review and editing. DH and DD: funding acquisition, supervision, and writing–review and editing. KM and J-PM: resources, supervision, and writing–review and editing. KO: funding acquisition and writing–review and editing. DR and CQ: resources, supervision, data curation, and writing–review and editing. TT: supervision and writing–review and editing. RV: data curation and writing–review and editing. GH: supervision, data curation, and writing–review and editing. DL and JF: writing–review and editing. LaM: supervision, conceptualization, methodology, and writing–review and editing. NN: funding acquisition, supervision, conceptualization, methodology, and writing–review and editing. All authors contributed to the article and approved the submitted version.
