# Supplementary material for: Gender, homelessness, hospitalization and methamphetamine use fuel depression among people who inject drugs: implications for innovative prevention and care strategies
Source: Front Psychiatry. 2023 Nov 1;14:1233844. doi: 10.3389/fpsyt.2023.1233844 (PMC10661402; doi:10.3389/fpsyt.2023.1233844)
Supplement: Supplementary file 1 [file Table_1.pdf]

Supplementary Table 1. Table 1: Comparison of participants characteristics at inclusion based on their dropout status.

**Characteristics**	**Staying (n=1142)**	**Dropped out (n=329)**	**p-value**
Sociodemographic			
Gender (male)	1062 (93.0)	307 (93.3)	0.84
**Age**	41.0 (± 7.9)	39.4 (± 8.9)	**<0.01**
At least High school level	705 (61.7)	207 (62.9)	0.70
**In a couple relationship**	473 (41.4)	102 (31)	**<0.01**
Income ⩾ 6 VND[1]	469 (41.1)	142 (43.2)	0.50
**Stable housing**	1111 (97.3)	313 (95.1)	**0.05**
Health insurance card	176 (15.4)	44 (13.4)	0.07
Sex work	11 (0.1)	3 (0.9)	0.72
Psychiatric data			
Anxiety at inclusion	89 (7.8)	33 (10)	0.19
**Depression at inclusion** (PHQ-2)	90 (7.9)	42 (12.8)	**0.01**
Suicidal ideation in past 2 weeks	75 (6.6)	10 (3.0)	0.35
Drugs habits			
Alcohol misuse (Audit-c)	439 (38.4)	136 (41.3)	0.31
**Methamphetamine > once a week**	193 (16.9)	74 (22.5)	**0.02**
Methamphetamine recent use[2]	740 (64.8)	227 (69.0)	0.13
Polydrug use[3]	162 (14.2)	56 (17.0)	0.20
> 10 years of heroin injection	328 (28.7)	79 (24.0)	0.09
Age of first heroin injection	29.5 (± 8.4)	29.4 (± 8.5)	0.76
High frequency heroin use	844 (74)	260 (79)	0.06
**Methadone Maintenance therapy**	289 (41.3)	46 (26.4)	**<0.01**
Infection status			
**HIV serology**	562 (49.2)	137 (41.6)	**0.01**
**Undetectable HIV**[4]	993 (87)	269 (82)	**0.04**
HCV serology	829 (72.6)	224 (68.1)	0.11

[1]Equivalent to ~260 USD per month; [2]In the month; [3]Cannabis, cocaine, ketamine or ecstasy; [4]Undetectable or viral load lower than 20 copies/mL. The results are given as numbers (percentage) or mean (± standard deviation).
