# Supplementary figures and images for: Gender, homelessness, hospitalization and methamphetamine use fuel depression among people who inject drugs: implications for innovative prevention and care strategies
Source: Front Psychiatry. 2023 Nov 1;14:1233844. doi: 10.3389/fpsyt.2023.1233844 (PMC10661402; doi:10.3389/fpsyt.2023.1233844)

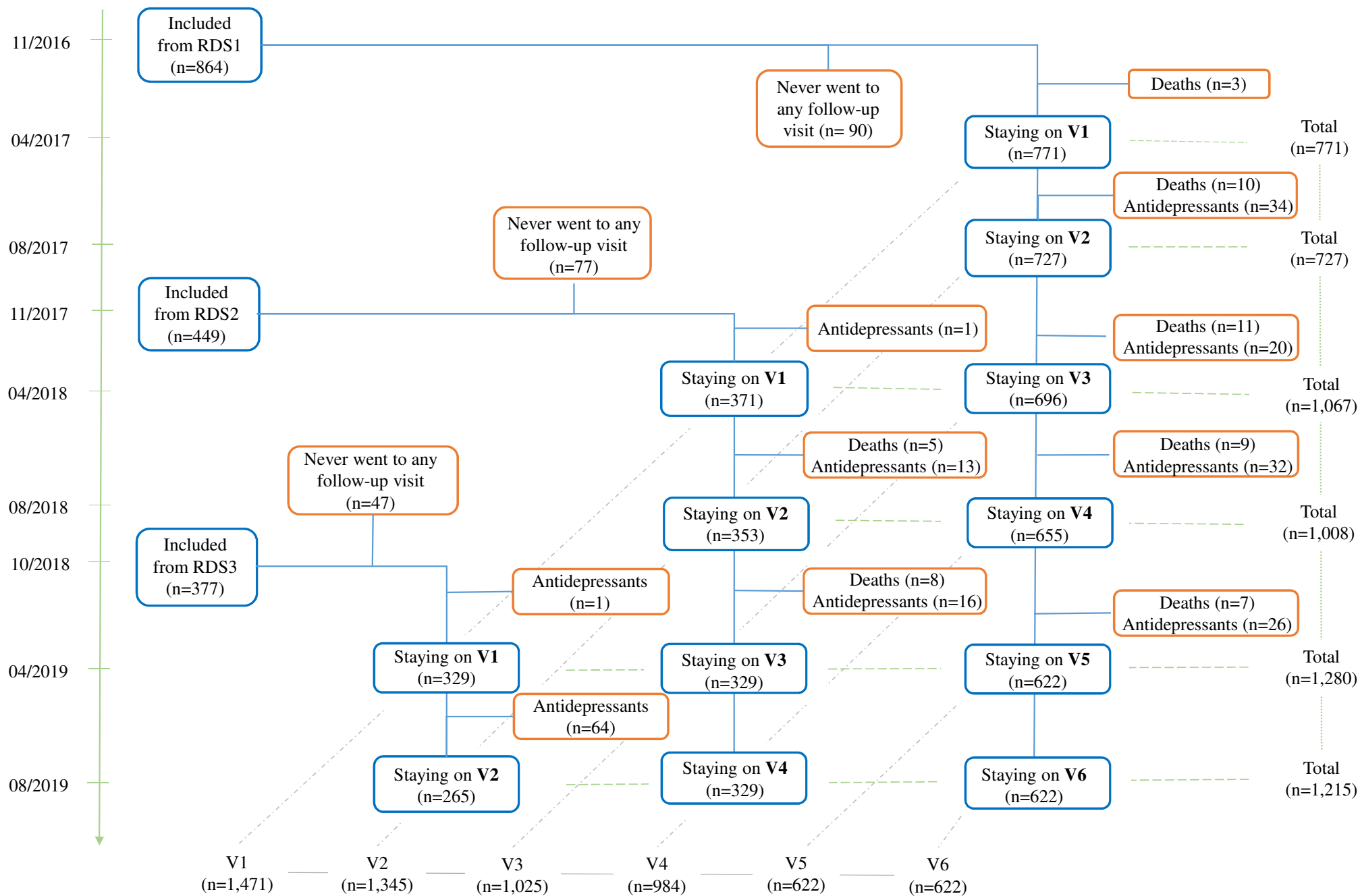

Supplementary Figure 1: Detailed Flow-chart

Supplement: Supplementary file 2 [file Image_1.pdf]
